# Supplementary material for: Group I introns in tRNA genes of Patescibacteria
Source: mSystems. 2026 Jan 30;11(3):e01536-25. doi: 10.1128/msystems.01536-25 (PMC13011429; doi:10.1128/msystems.01536-25)
Supplement: Supplemental material — Figures S1-S3 and legends for Tables S1-S10. [file msystems.01536-25-s0001.pdf]

Supplementary Materials

Group I Introns in tRNA Genes of Patescibacteria

Yuna Nakagawa[1,2], Kazuaki Amikura[2], Kimiho Omae[2], Shino Suzuki[2,3]#

[1]Department of Integrated Biosciences, Graduate School of Frontier Sciences, The University of Tokyo, Kashiwa, Chiba, Japan
[2]Geobiology and Astrobiology Laboratory, RIKEN Pioneering Research Institute, Wako, Saitama, Japan
[3]Institute of Space and Astronautical Science (ISAS), Japan Aerospace Exploration Agency (JAXA), Sagamihara, Kanagawa, Japan

Running Head: Introns in Patescibacteria tRNA

#Address correspondence to Shino Suzuki, shino.suzuki@riken.jp

Supplementary Tables (provided as a separate file)

Table S1. List of bacterial genomes registered as complete genomes in GTDB r220 used in this study

Table S2. Detection results of tRNA genes corresponding to the 20 canonical amino acids in complete genomes of Patescibacteria by tRNAscan-SE 2.0, ARAGORN, and tFind

Table S3. Information on tRNA sequences containing group I introns in Patescibacteria

Table S4. Sequences used for in vitro splicing assays

Table S5. Information on tRNA sequences containing group I and group II introns

Table S6. Information on homing endonucleases and reverse transcriptases encoded within intron-containing tRNAs

Table S7. Group I introns predicted by Infernal (E-value < 1E-4) from bacterial genomes

Table S8. Group II introns predicted by Infernal (E-value < 1E-4) from bacterial genomes

Table S9. Information on tmRNA sequences containing group I introns

Table S10. Information on protein sequences used to build the BLASTx database for CDS prediction, downloaded from reviewed entries

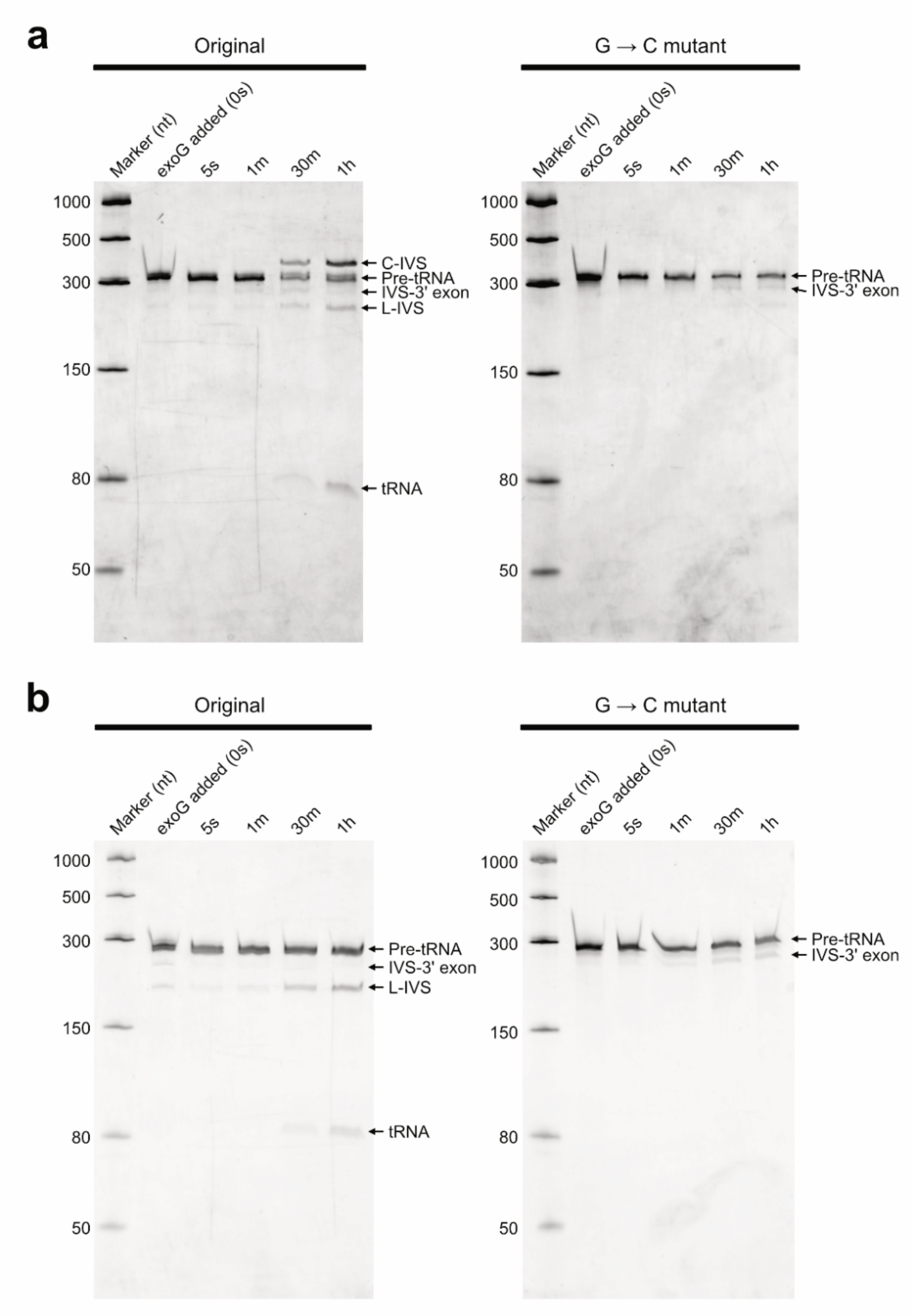

Fig S1. Splicing activity of group I introns inserted at position 35/36 in tRNA genes of Patescibacteria

(a) Gel electrophoresis of splicing reactions of the $tRNA^{Asn}$ containing a group I intron from the Patescibacteria genome (accession: GCA_016700035.1).

(b) Gel electrophoresis of splicing reactions of the $tRNA^{Asp}$ containing a group I intron from the Patescibacteria genome (accession: GCA_001029755.1).

(a-b) Time points after GTP addition are indicated above each lane (0 seconds, 5 seconds, 1 minute, 30 minutes and 1 hour). Both original constructs based on the genome sequences and mutant constructs (3' terminal G→C substitution in the group I intron) are shown. For each tRNA gene, the original and mutant constructs were assayed at the same concentration and loaded on the same gel.

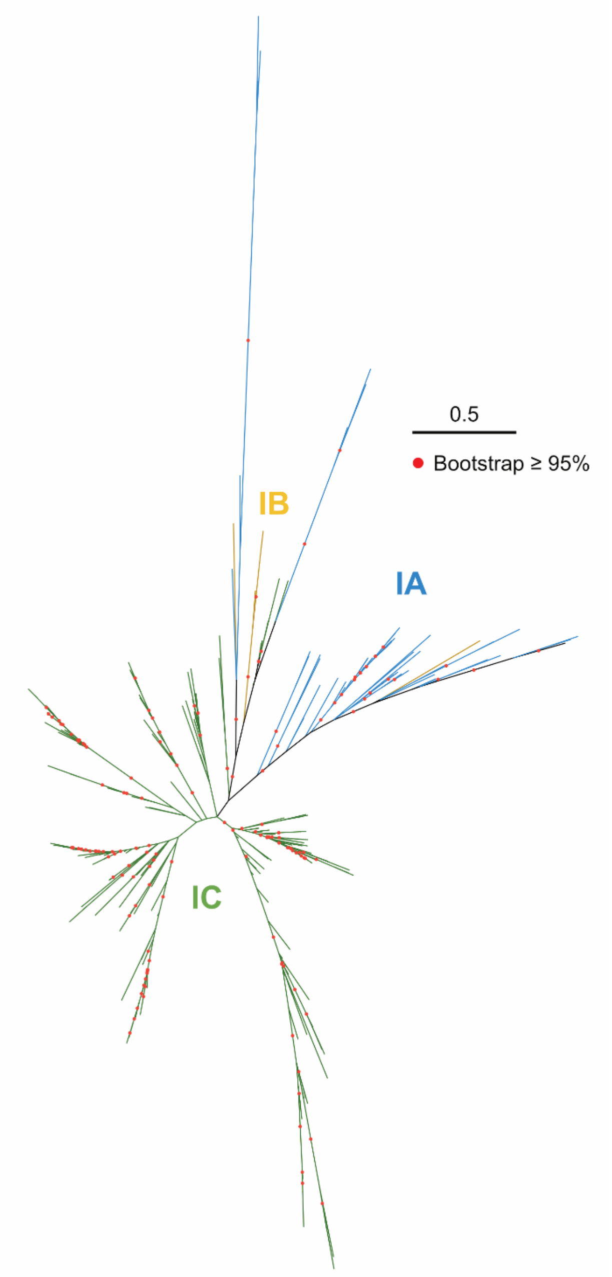

Fig S2. Maximum-likelihood phylogenetic tree of 333 group I intron sequences inserted into bacterial tRNA genes

The tree was inferred using IQ-TREE with the TIM2e+R6 model selected by ModelFinder, based on 152 nucleotide sites. The bootstrap values ≥ 95% are indicated by red dots. Blue branches indicate the IA subgroup, yellow branches indicate the IB subgroup, and green branches indicate the IC subgroup.

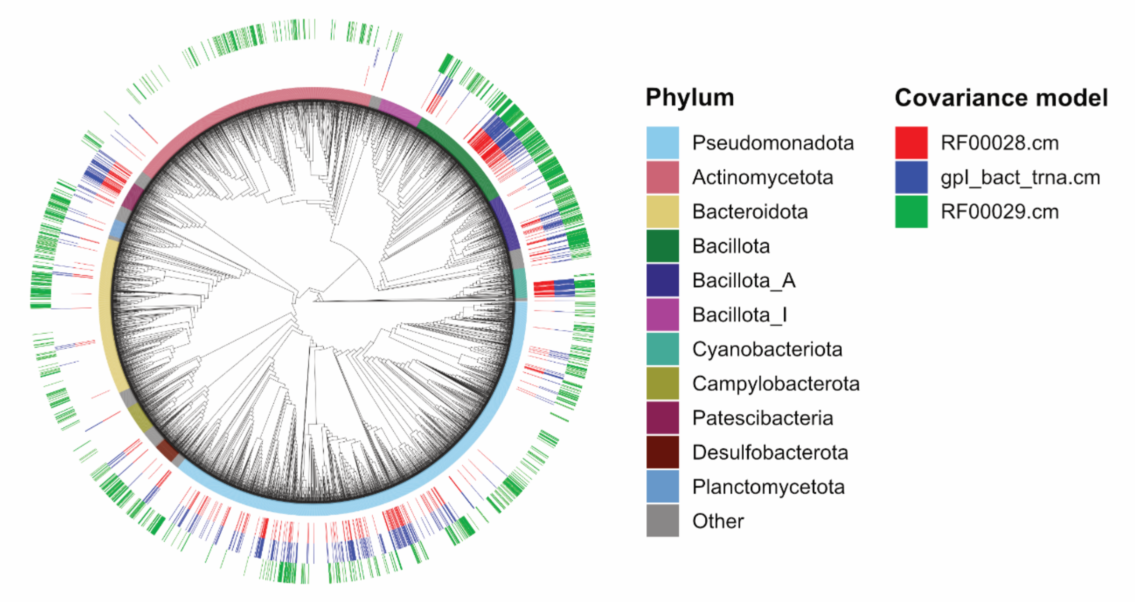

Fig S3. Phylogenetic distributions of group I and group II introns across bacterial genomes

Phylogenetic tree of 4,934 bacterial genomes constructed using concatenated alignments of bac120 marker genes. The tree was inferred using IQ-TREE with the Q.pfam+R10 model selected by ModelFinder, based on 5,035 amino acid positions. Phylum and intron detection results based on covariance models (group I intron: RF00028.cm and gpI_bact_trna.cm; group II intron: RF00029.cm) were mapped on the tree.
